# From the Gut to the Liver: Another Organ to Watch in FAP Patients

**DOI:** 10.1155/2016/1738696

**Published:** 2016-03-24

**Authors:** Spencer Paulson, Charmi Patel, Hitendra Patel

**Affiliations:** ^1^Department of Pathology, University of Arizona, Tucson, AZ 85724, USA; ^2^Section of Hematology and Oncology, University of Arizona, Tucson, AZ 85724, USA

## Abstract

We report a rare association of hepatocellular carcinoma with familial adenomatous polyposis in a young patient and its clinical significance. A 28-year-old female with a past medical history of familial adenomatous polyposis (FAP) and subsequent colonic adenocarcinoma underwent total colectomy. She later presented with intermittent right upper quadrant pain and nausea of four months' duration. MRI of the abdomen revealed multiple liver lesions, the largest 8.5 cm in diameter, with radiologic features suggestive of hepatocellular carcinoma. A CT-guided liver biopsy demonstrated well-differentiated HCC which was confirmed by immunohistochemistry. In patients with a history of FAP, a heightened awareness of the possibility of concurrent or subsequent HCC is warranted.

## 1. Introduction

Familial adenomatous polyposis (FAP) is an autosomal dominant disease occurring in approximately 1 out of 10,000 to 1 out of 30,000 live births. FAP is caused by a mutation in the adenomatous polyposis coli (APC) gene which is involved in the Wnt/APC/Beta-catenin pathway. Affected individuals exhibit hundreds to thousands of colorectal adenomas in the second to third decade of life, with nearly a 100% risk of colorectal cancer if left untreated. Extracolonic manifestations of FAP include adenomas and carcinomas of the small intestine, stomach, and biliary tract; osteomas of the mandible, skull, and long bones; soft tissue lesions such as epidermoid cysts and desmoid tumors; thyroid carcinomas; and central nervous system neoplasms [1, 2]. It is rare for patients with FAP to have hepatic neoplasms [3]. However, hepatic tumors reported in FAP patients include adenoma, hepatoblastoma, fibrolamellar carcinoma, and HCC [4–6]. Here we report a case of HCC in a young patient with FAP.

## 2. Case Presentation

A 28-year-old African American female presented with intermittent right upper quadrant pain and nausea of four months' duration. The pain was a dull ache, radiating to her back and worsening progressively over several weeks. Her past medical history was significant for familial adenomatous polyposis (FAP) status-post total colectomy after diagnosis of colonic adenocarcinoma at age 12. She did not have any recurrence of her colon cancer. She denied any history of ethanol abuse, blood transfusion, trauma, fever, weight loss, emesis, diarrhea, constipation, melena, heartburn, or shortness of breath. She had a family history of FAP in her father, three paternal aunts, and two sisters. Her paternal grandmother died of colon cancer at age 60. Physical examination revealed a round, tender, nondistended abdomen, with positive Murphy's sign, normal bowel sounds, and no hepatosplenomegaly, jaundice, or icterus.

Laboratory studies revealed mildly elevated liver enzymes with alkaline phosphatase of 469, alanine aminotransferase of 175, aspartate aminotransferase of 170, normal bilirubin, total protein of 7.5, and albumin of 3.1. Her serum alpha fetoprotein (AFP) level was not elevated. Serologic studies for hepatitis A, hepatitis B, and hepatitis C were negative.

**Figure 1:**
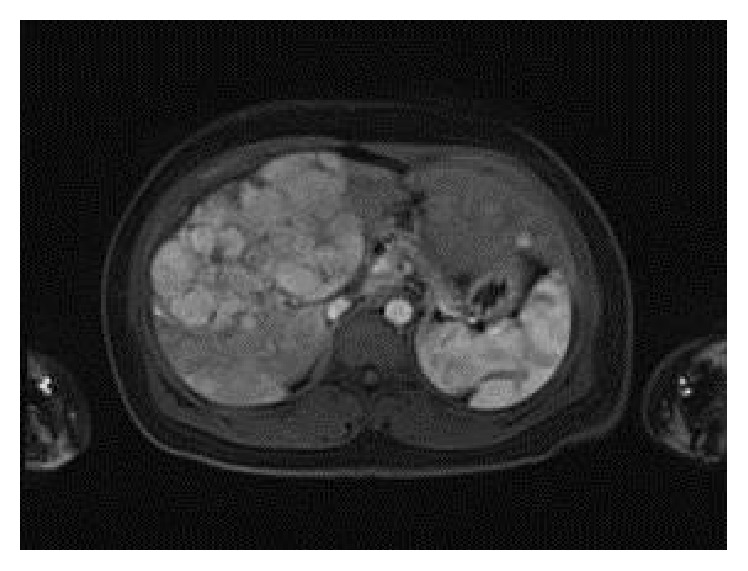
MRI of the abdomen with contrast shows multiple lesions in the right and left liver lobes, the largest 8.5 cm.

**Figure 2:**
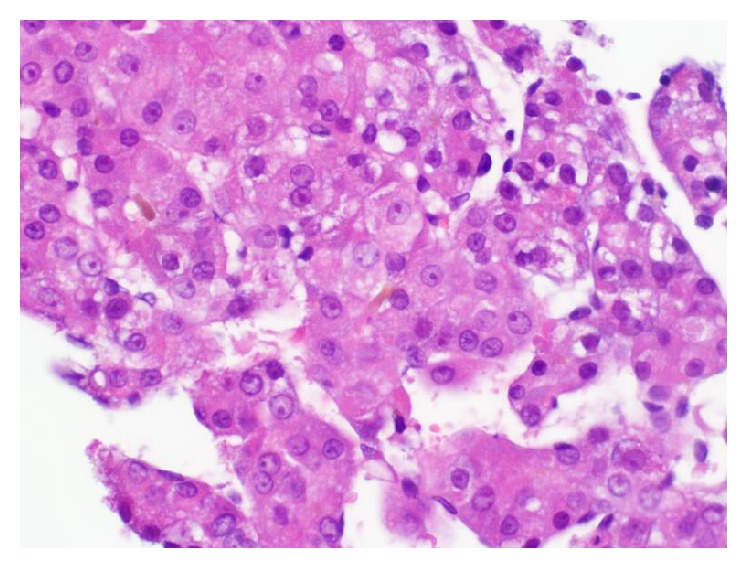
Hematoxylin- and eosin-stained section of a CT-guided core biopsy of the liver lesion shows well-differentiated to moderately differentiated hepatocellular carcinoma in a trabecular pattern (40x magnification).

**Figure 3:**
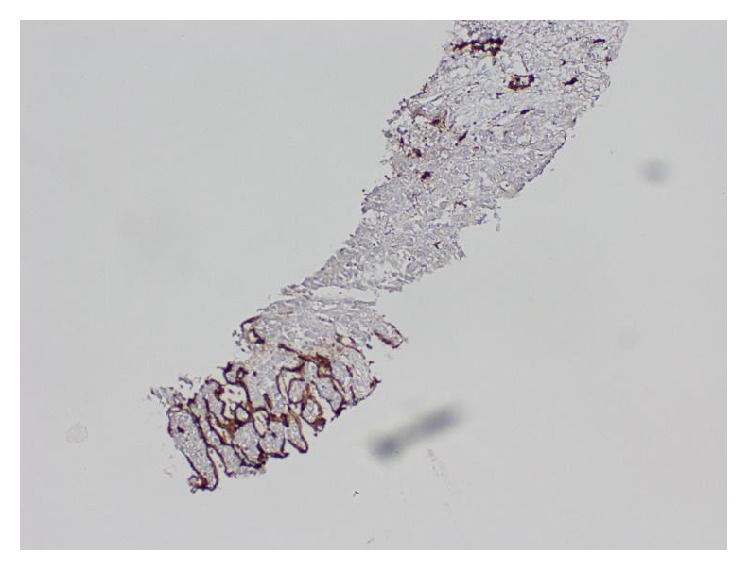
Immunohistochemical stain for CD34 performed on a core needle biopsy of the liver lesion highlights endothelial cells surrounding tumor cells (10x magnification).

Ultrasound of the abdomen demonstrated a nodular liver surface with multiple heterogeneous nonspecific hyperechoic liver lesions. MRI of the abdomen with and without contrast revealed multiple lesions, the largest 8.5 cm in diameter, scattered in the left and right lobes of the liver, exhibiting arterial enhancement, washout in the delayed phase, and signs of internal hemorrhage, consistent with hepatocellular carcinoma (HCC) (Figure 1). A CT-guided liver biopsy was performed. Hematoxylin- and eosin-stained sections showed well-differentiated HCC. Tumor cells were polygonal with prominent nuclei. No portal tracts were present in the area of tumor. Tumor cells were arranged in trabecular and pseudoacinar patterns with some gland-like spaces containing bile material (Figure 2). Background liver was unremarkable with no fibrosis. The diagnosis of HCC was confirmed by immunohistochemistry. CD34 highlighted endothelial cells surrounding trabeculae (Figure 3). Polyclonal CEA highlighted the canalicular membrane. HepPar-1 was strongly positive in both tumor and normal liver, effectively excluding the possibility of liver metastasis from another primary site.

The patient underwent trans-catheter arterial chemoembolization and was started on sorafenib, which initially was poorly tolerated, with significant nausea. However, with continued treatment there was complete resolution of left lobe lesions and partial response of lesions in the right lobe. She continued to have severe abdominal pain with nausea and vomiting. She was noncompliant to follow-up in clinic at that point and, per a telephone report, she developed lung metastases. Since that time she was lost to follow-up.

## 3. Discussion

Cases of liver malignancy in subjects with FAP have been described in rare case reports [4–6]. Cases of HCC in FAP patients suggest that the Wnt/APC/Beta-catenin pathway, of which APC is but one member, may be important in hepatocarcinogenesis in general [7]. Increased Beta-catenin appears to be a key event in HCC carcinogenesis. Mutations in the Beta-catenin gene have been found in 17% of HCC cases, and 50–70% of HCC cases have increased levels of Beta-catenin in the cytoplasm and nucleus [8]. Although increased Beta-catenin can be seen in HCC due to APC inactivation, there are other documented causes as well, including hepatitis C infection, increased cell turnover, and mutation in other proteins involved in the molecular pathway [9]. These observations suggest that mutations in the Wnt/APC/Beta-catenin pathway are necessary pathologic events in both FAP and HCC.

The interactions among Wnt, APC, and Beta-catenin have been well characterized. The Wnt protein binds to a receptor named Frizzled, which in turn upregulates a downstream effector protein Dishevelled, and eventually interacts with the APC protein complex [10]. APC is considered a tumor suppressor protein that forms a complex with glycogen synthase kinase 3-beta (GSK-3Beta), as well as axin. The APC complex binds to casein kinase 1- (CK1-) phosphorylated Beta-catenin, and the GSK-3Beta component of the complex phosphorylates the Beta-catenin a second time, ultimately leading to its ubiquitination and degradation by proteasomes. Loss of APC function, therefore, leads to stabilization and accumulation of Beta-catenin in the cytoplasm and nucleus, where it binds to the transcription factor Tcf/Lef complex and activates the transcription of Wnt target genes. Approximately 150 Wnt target genes have been identified, including MYC, MYB, CJUN, and CYCD1, which play pivotal roles in cell proliferation, differentiation, migration, and interaction with the extracellular matrix. Due to the limited amount of tissue available, immunohistochemical staining for Beta-catenin was not possible in our case.

If similar genetic alterations underlie both FAP and HCC, then we would expect to see more cases of the two diseases arising in the same patient. In FAP, only one of the two APC alleles has been inactivated, and a “second hit” that inactivates the other, normal allele is required for neoplastic development. The rarity of HCC in FAP patients may simply be due to a lower rate of mutation in hepatocytes when compared with colonic epithelial cells. Alternatively, there may be a substantial difference in response to APC inactivation by hepatocytes versus colonic epithelial cells. Perhaps APC mutations are necessary but not sufficient for HCC development. In this scenario, additional genetic “hits” may be required before HCC can develop, even in the context of preexisting APC mutations.

This case demonstrates a rare but plausible association of FAP and hepatocellular carcinoma in a young patient with no other predisposing risk factors for HCC. Some genetic abnormalities found in both APC and HCC involve the same molecular pathway, (Wnt/APC/Beta-catenin). In patients with a history of FAP, a heightened awareness of the possibility of concurrent or subsequent HCC is warranted.
